# The Use of Stem Cell-Derived Neurons for Understanding Development and Disease of the Cerebellum

**DOI:** 10.3389/fnins.2018.00646

**Published:** 2018-09-26

**Authors:** Samuel P. Nayler, Esther B. E. Becker

**Affiliations:** Department of Physiology, Anatomy and Genetics, University of Oxford, Oxford, United Kingdom

**Keywords:** cerebellum, stem cell, organoid, differentiation, ataxia, neuronal, Purkinje cell, granule cell

## Abstract

The cerebellum is a fascinating brain structure, containing more neurons than the rest of the brain combined. The cerebellum develops according to a highly orchestrated program into a well-organized laminar structure. Much has been learned about the underlying genetic networks controlling cerebellar development through the study of various animal models. Cerebellar development in humans however, is significantly protracted and more complex. Given that the cerebellum regulates a number of motor and non-motor functions and is affected in a wide variety of neurodevelopmental and neurodegenerative disorders, a better understanding of human cerebellar development is highly desirable. Pluripotent stem cells offer an exciting new tool to unravel human cerebellar development and disease by providing a dynamic and malleable platform, which is amenable to genetic manipulation and temporally unrestricted sampling. It remains to be seen, however, whether *in vitro* neuronal cultures derived from pluripotent stem cells fully recapitulate the formation and organization of the developing nervous system, with many reports detailing the functionally immature nature of these cultures. Nevertheless, recent advances in differentiation protocols, cell-sampling methodologies, and access to informatics resources mean that the field is poised for remarkable discoveries. In this review, we provide a general overview of the field of neuronal differentiation, focusing on the cerebellum and highlighting conceptual advances in understanding neuronal maturity, including a discussion of both current and emerging methods to classify, and influence neuroanatomical identity and maturation status.

## Introduction

The development of the nervous system is guided by temporally programmed, spatially distinct morphogen gradients. *In vitro* reproduction of these cues can be achieved in pluripotent stem cells (PSCs), facilitating cellular differentiation into distinct neuronal and glial subtypes (Zhang et al., 2001; Wichterle et al., 2002). The advent of induced PSC (iPSC) technology has greatly advanced the field by enabling the *in vitro* derivation of PSCs from almost any somatic cell type. This has allowed for the generation of iPSCs from patients and thus linkage of *in vitro* cellular phenotypes to distinct clinical presentations. Over recent years, enormous progress has been made in differentiating patient and control PSCs into cortical (Brennand et al., 2015), motor (Dimos et al., 2008; Sances et al., 2016), and dopaminergic (Woodard et al., 2014; Sandor et al., 2017) neurons. Moreover, three-dimensional (3D) PSC-derived organoid cultures offer exciting possibilities to study brain development, evolution, and abnormalities that underlie developmental disorders (Kelava and Lancaster, 2016; Camp and Treutlein, 2017).

Despite these technological breakthroughs, the potential of PSC-derived neuronal models remains far from fully realized. Current challenges include the development of standardized, robust protocols for differentiation, as well as classification strategies that can effectively relate *in vitro*-derived cell types to their *in vivo* counterparts, including assessing their state of maturity. Thus far, PSC-derived models have mainly been utilized to explore very early stages of brain development associated with disorders, including correct neural tube polarization and apico-basal polarity establishment (Yoon et al., 2014), microcephaly (Lancaster et al., 2013), and Zika-related viral infection (Dang et al., 2016; Garcez et al., 2016; Qian et al., 2016). PSC-derived neuronal cells are usually not exposed to the same range of environmental stimuli, which aid in the development of neurons within the correct physiological context of the developing brain *in vivo*. It therefore remains to be established whether PSC-derived neuronal models are able to recapitulate late developmental events including the formation of complex cellular interactions and neuronal networks. Finally, the degree to which a given disease can be recreated in synthetic *in vitro* environments remains an important consideration.

Methodologies to generate cerebellar neurons from human PSCs and to model cerebellar disorders are beginning to emerge (Muguruma et al., 2015; Ishida et al., 2016; Sundberg et al., 2018; Watson et al., 2018). Here, we review the current state of the field and highlight conceptual advances that will help to establish relevant model systems for the study of cerebellar development and disease using PSCs.

## Recapitulation of Cerebellar Ontogenesis *In Vitro*

**FIGURE 1:**
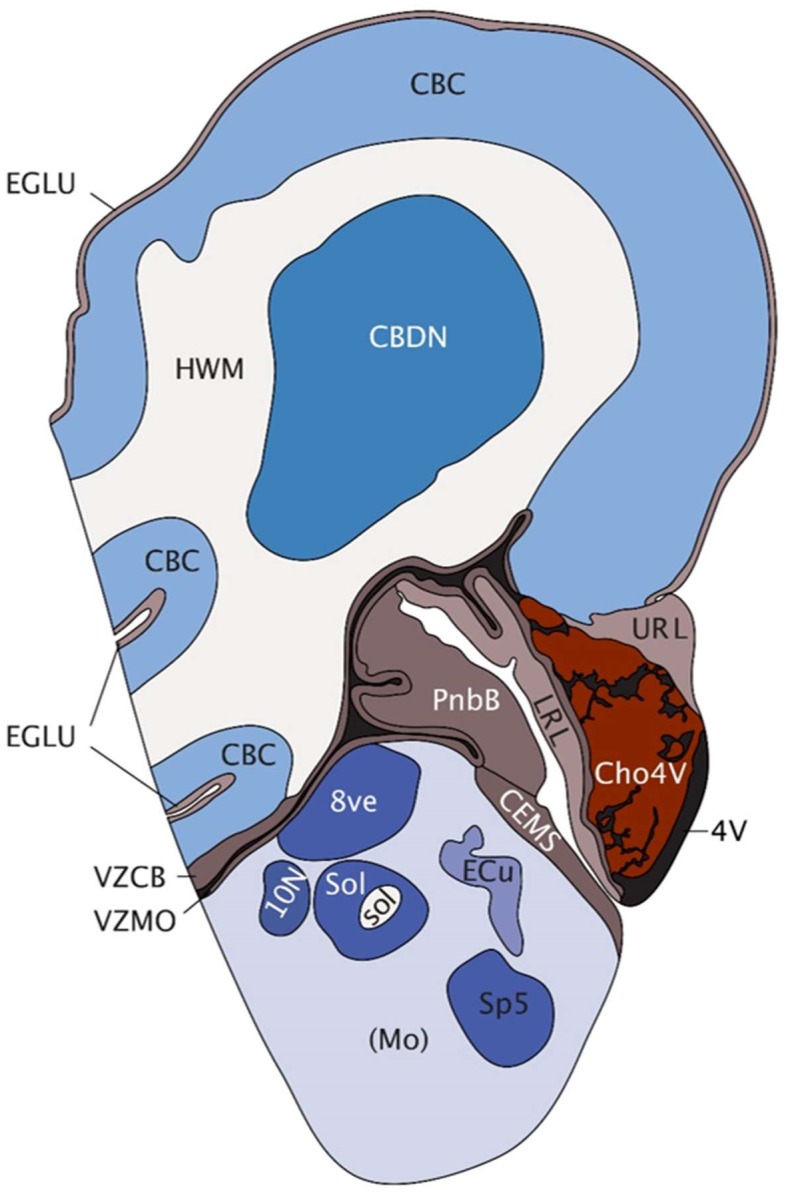
Saggital depiction of human hindbrain at post-conception week 15. EGLU, external granule layer; CBC, cerebellar cortex; HWM, white matter of hindbrain; CBDN, cerebellar deep nuclei; 4V, fourth ventricle; VZCB, ventricular matrix zone of cerebellum; VZMO, ventricular matrix zone of medulla; URL, upper rhombic lip; LRL, lower rhombic lip; Cho4V, choroid plexus of the fourth ventricle; PnbB, pontobulbar body; CEMS, caudal (posterior) extramural migratory stream; Mo, medulla oblongata; 8ve, vestibular nuclei in medulla; 10N [(dorsal motor nucleus of the vagus, vagal nucleus)], Sol, solitary nucleus; sol, solitary tract; Ecu, external cuneate nucleus; Sp5, spinal trigeminal matrix. Image taken with permission from the Allen Brain Atlas (http://atlas.brain-map.org) [Accessed 2018].

The cerebellum is one of the first brain structures to emerge and develops over a long period of time until the first postnatal years (Wang and Zoghbi, 2001). Following generation of the neural plate and tube, the developing embryonic brain begins to organize into three distinct compartments, the prosencephalon, mesencephalon, and rhombencephalon (Hatten and Heintz, 1995; Wassef, 2013). Development of the cerebellar anlage is dependent on fibroblast growth factor (FGF) signaling from the isthmic organizer, which is located at the mid-hindbrain boundary (MHB) and demarcated by the expression of distinct transcription factors including OTX2, GBX2, EN1/2, and PAX2 (Wang and Zoghbi, 2001; Butts et al., 2014; Leto et al., 2016). Unique to cerebellar development, progenitors are generated in two distinct germinal zones in rhombomere 1; the ventricular zone (VZ) gives rise to GABAergic neurons [Purkinje cells (PCs), interneurons, GABA-ergic cerebellar nuclei neurons], whereas all glutamatergic neurons [granule cells (GCs), unipolar brush cells, glutamatergic cerebellar nuclei neurons] are generated from the rhombic lip (RL) (Hoshino et al., 2013; Butts et al., 2014; Leto et al., 2016). Embryonic and postnatal cerebellar development are driven by both symmetric and asymmetric division and migration of progenitors and subsequent neuronal differentiation, ultimately giving rise to a highly organized structure containing more neurons than the rest of the brain combined. In the human brain, 80% of the total number of neurons in the brain, i.e., approximately 69 billion neurons, are found in the cerebellum (Herculano-Houzel, 2009). By week 15 of development, the human cerebellum already shows remarkable complexity (**Figure 1**).

Early studies using mouse embryonic stem cells (ESCs) aimed to recapitulate the signals that occur during *in vivo* cerebellar development for the *in vitro* differentiation of cerebellar neurons. Initial studies focussed on the stepwise addition of growth factors and mitogens including FGF8 and retinoic acid (RA) to initiate cerebellar patterning and differentiation (Salero and Hatten, 2007). Notably, medium conditioned by primary cultured cerebellar cells improved survival and induced the expression of mature markers in differentiated ESC-derived cerebellar cultures.

Su et al. (2006) pioneered an approach utilizing serum-free culture of embryoid body-like aggregates (SFEB) in combination with dorsalizing activity of bone morphogenetic protein (BMP) signaling. This approach gave rise to progenitors of both germinal zones (RL and VZ), including ATOH1/MATH1-positive GC precursors and cerebellar neurons expressing the PC markers L7/PCP2 and Calbindin.

The SFEB methodology was subsequently refined, yielding a 30-fold improvement in PC production, ostensibly due to the more precise way in which the conditions resembled the local self-inductive signaling events operational during development of the cerebellum *in vivo* (Muguruma et al., 2010). Additionally, this study advanced understanding of both FGF2 and Insulin as caudalizing factors assisting in MHB pre-patterning. 75% of neural rosettes expressed the cell-surface marker Kirrel2/NEPH3, a downstream target of PTF1α, which is expressed in all VZ progenitors (Leto et al., 2016). This allowed isolation by flow cytometry and expansion of a purified population of PC progenitors. Importantly, this study also showed orthotopic integration of ESC-derived PCs into the mouse cerebellum following *in utero* electroporation into the sub-ventricular space at E15.5 (Muguruma et al., 2010). Remarkably, these PCs exhibited engraftment, proper cell polarity, and projections as well as expression of synaptic markers. Whilst surviving transplantation, purified Kirrel2-positive cells could not be maintained alone *in vitro*, but required co-culture with RL-derived GCs for their further differentiation, and maturation. This is consistent with other studies reporting a drastic improvement in ESC-derived PC generation through co-culture with dissociated cerebellar cultures or organotypic slice cultures of whole mouse cerebellum (Tao et al., 2010), and highlights the need for an appropriate trophic environment for the *in vitro* culture of neuronal subpopulations.

More recent studies have further developed these protocols for the differentiation of cerebellar neurons derived from human PSCs. Based on the method by Salero and Hatten (Salero and Hatten, 2007), Erceg et al. (2010) treated embryoid bodies (EBs) aggregated from hESCs with FGF8b and RA, followed by a multi-stage-specific application of growth factors and mitogens. This protocol utilized a manual selection process to isolate polarized neuroepithelium and extended a number of the stages, yielding cerebellar cells expressing markers of GCs, PCs, and glial cells. A subsequent transcriptomic analysis on iPSCs differentiated according to this protocol showed that their transcriptomic signature most closely resembled the human fetal cerebellum at 22 weeks of development, when compared to temporally and spatially discrete regions of the human brain (Nayler et al., 2017).

Ishida et al. (2016) applied their earlier-developed mouse ESC protocol for the generation of cerebellar neurons from human ESCs and subsequently human iPSCs. By day 35 approximately 28% of cells expressed Kirrel2, forming rosettes that resembled polarized neuroepithelial tissue. Unlike the murine protocol, exogenous inhibition of ventralizing Hedgehog signals with cyclopamine was not required for the specification of human Kirrel2-positive cells. However, similar to the murine protocol, Kirrel2-positive cells required co-culture with murine RL-derived cerebellar cultures to promote survival and further differentiation. This suggests that there are factors necessary for long-term growth and maturation of human PCs that are still unknown. Nevertheless, this protocol marks a major conceptual leap and offers a more economically feasible protocol for the differentiation of human cerebellar cells than previously available, with fewer sources of experimental variability. Recently, an adaptation of the protocol was reported that eliminates sorting of Kirrel2-positive cells and employs co-culture of E18.5 mouse cerebellar progenitors rather than RL-derived cultures (Watson et al., 2018). As early as day 35 of differentiation, subpopulations of iPSC-derived cells expressed markers of the two cerebellar germinal zones. Calbindin-positive PC progenitors were detected from day 50 onward with 10% of human cells staining positive by day 70 of differentiation.

Another approach made use of human iPSC-EBs that were treated early with FGF2, Insulin, and cyclopamine, resulting in 10% of cells expressing Kirrel2 after 20 days (Wang et al., 2015). Further maturation of isolated Kirrel2-positive cells was achieved through co-culture with rat organotypic slices (P9–10). However, significant electrophysiological activity of the iPSC-derived neurons was observed only following co-culture with human fetal cerebellar slices (16–23 post-conception weeks).

More recently, an alternative protocol was published using both FGF8b and FGF2 to instigate cerebellar patterning (Sundberg et al., 2018). Greater yields (61–91%) of maturing PCs, positive for L7/PCP2, were obtained using immunopanning for the specific cell surface antigen Thy-1 instead of cell sorting for Kirrel2. Cultures were maintained for up to 140 days in the presence of postnatal mouse GCs and displayed expression of mature synaptic markers and electrical activity.

Most of the published protocols to date focus on the generation of PCs from human PSCs, while the differentiation into other cerebellar cell types remains relatively unexplored. The addition of FGF19 and SDF1 to SFEB cultures has been reported to promote the spontaneous generation of polarized neural tube-like structures with a three-layer cytoarchitecture reminiscent of the embryonic cerebellum (Muguruma et al., 2015). This suggests that human ESC-derived cerebellar progenitors show significant potential for self-organization. However, the potential of organoid cultures to recapitulate the full cerebellar ontogenesis remains to be further explored.

Another challenge remains the long-term culture and maturation of human PSC-derived cerebellar neurons without the presence of mouse co-cultures. Mature phenotypes of PSC-derived PCs have so far only been demonstrated in co-culture or, more convincingly, by transplantation of differentiated cells into mouse cerebellum, where signaling factors, and the local micro-environment coax them toward terminal differentiation and/or integration with the host cerebellar circuitry (See **Supplementary Table 1** for summary). While this demonstrates the potential of the PSC-derived neurons to mature into functioning cerebellar neurons, it also highlights the need to better understand the factors that promote the maturation of PSC-derived cerebellar neurons. A growing number of methods exist for reverse-engineering specific cellular micro-environments and the cells and molecules which constitute these (Murrow et al., 2017). It is likely that the combination of these technologies will be instrumental in elucidating key conditions that promote long-term survival and maturation of PSC-derived cerebellar neurons. These methodologies might also be exploited to identify factors that could increase the neurogenic potential of the mature cerebellum, which is believed to be one of the most static structures in the brain (Ponti et al., 2008).

## Emerging Technologies for Engineering Neural Stem Cell Micro-Environments

When modeling brain development *in vitro*, one should be cognizant of the trade-off between purity and complexity of neuronal cultures (Kelava and Lancaster, 2016), with ‘pure’ cultures representing a facile state that does not exist in nature. For example, a pure population of PCs, while facilitating stringent biochemical analyses, would not be informative in terms of modeling developmental and physiological processes that are dependent on the interaction with other neurons, or glial cells. Physiologically relevant insight will therefore likely require recapitulation of local niches, including the presence and interactions of astrocytes, neurons, microglia, and potentially vasculature. Such an approach has been pioneered by combining neural precursors, endothelial cells, mesenchymal stem cells and microglia/macrophage progenitors in chemically defined polyethylene-glycol hydrogels (Schwartz et al., 2015). This exemplified a model system comprising a milieu of independently engineered cell types, striving to recapitulate the complex interplay of these cells and their collective response to a range of compounds as a method for drug screening and discovery. Moreover, 3D printing of cells will be helpful to reconstruct the spatial relationship of different cell populations (Graham et al., 2017; Gu et al., 2017; Yanagi et al., 2017). The potential for isolation by bead-sorting or flow cytometry and recombination in a controlled deposition by 3D printing makes it technically feasible to generate cellular constructs in which the organization of cells is specified by the user. In addition, the use of bioengineering approaches to recreate physiologically relevant chemical signaling gradients or varied physical parameters such as surface tension may be advantageous. Prime examples would be the recapitulation of concentration gradients of Sonic Hedgehog or Reelin, known to direct cerebellar migration, and patterning. Such an approach would be aided by the use of devices, such as bioreactors featuring cells in serial array, to allow the study of paracrine/autocrine signaling (Titmarsh et al., 2016). Combined with genetic-reporters this allows combinatorial factor screening in order to monitor cell-fate commitment to systematically optimize culture conditions (Titmarsh et al., 2013).

**FIGURE 2:**
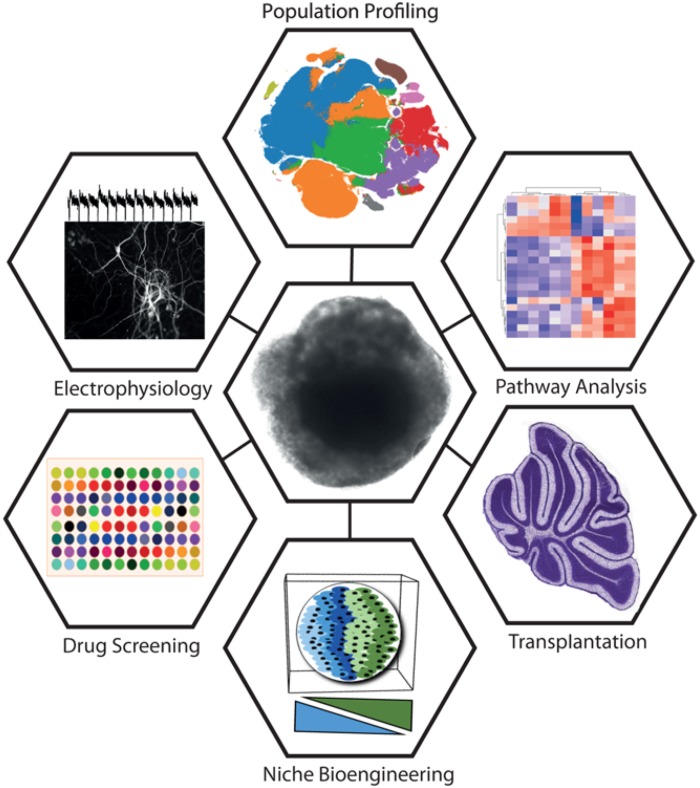
Summary of key approaches being utilized synergistically with organoid technology. A cerebellar organoid is shown in the center. Approaches clockwise from top: Rapid developments in single-cell sequencing technologies drive new methods of population profiling. Shown is a tSNE plot constructed from the 10X Million Cell Dataset (data accessible at NCBI GEO database, accession GSM2453144). Pathway analysis is a broad set of approaches commonly utilizing differential expression and gene-set enrichment analysis methods to identify coordinately dysregulated genes as representative of specific pathway perturbations. Transplantation offers a means to test cellular plasticity, lineage commitment, and factors required for terminal differentiation (figure depicting P14 mouse cerebellum has been adapted from the Allen Brain Atlas). Niche bioengineering allows the *in vitro* reconstruction of elements of the *in vivo* niche, using approaches such as 3D printing, hydrogels, microfabricated devices, and co-culture. Drug screening stands to benefit from organoids by providing physiologically relevant surrogate model systems for the organ of interest for high-throughput testing. Electrophysiology is a widely adopted tool for establishing neuronal maturity and connectivity (trace adapted from Nayler et al., 2017).

Organoids show extraordinary promise in recapitulating the complex architecture of the developing brain. An interesting recent example of this is the addition of Matrigel to the organoid culture environment, which appears to mimic the basement membrane (Lancaster et al., 2017). It will be intriguing to apply this to cerebellar organoids, where Laminin, β1-integrins and dystroglycans of the basement membrane have a distinct role in astroglial specialization (Nguyen et al., 2013; Leto et al., 2016). However, organoid technology faces several limitations surrounding the maturity reached following differentiation, intrinsic heterogeneity, and size constraints with oxygen and nutrient diffusion (Okkelman et al., 2017; Quadrato et al., 2017). The latter might be overcome using vascularized organoids (Mansour et al., 2018; Pham et al., 2018). The cerebellum is a prime candidate for this technology, given that it is one of the most highly vascularized sites in the brain. A recent development involved *in vitro* patterning and fusion of adjacent brain areas to mimic migration and long-range developmental interactions (Bagley et al., 2017). Approaches such as this may shed light on developmental disorders of the cerebellum involving abnormal migration and positioning of cerebellar precursor cells. A summary of key approaches that could be utilized synergistically with organoid technology is shown in **Figure 2**.

## Neuronal Classification Strategies Using Transcriptomic Data

Major efforts to identify molecular markers for discrete sub-populations of cerebellar cells at distinct points in time have come from studies of the mouse (Sato et al., 2008; Ha et al., 2012). An early example of this, GENSAT, made use of eGFP-BAC transgenic mice to isolate and identify patterns of epoch/cell-type specific gene expression (Heintz, 2004). While a number of genetic-reporters have been utilized to study differentiation and facilitate experimental handling, their widespread adoption in human systems has been slow. This may be explained by the notion that cell-type specific markers are often facile in the context of PSC differentiation, owing to non-equivalent time scales, species-specific ontological differences, and the lack of spatial congruence as a means for anatomical orientation. The rapid development of increasingly affordable expression-profiling strategies combined with powerful cell-sampling methodologies, including laser-capture-microdissection, cell-surface marker isolation by FACS, barcoding, and microfluidic capture of single cells have dramatically changed what is now experimentally possible.

Large data repositories are driving approaches to isolate spatio-temporal signatures that can be used to move to a fuller understanding of the neuroanatomical identity and maturity of human neurons following differentiation (Kang et al., 2011; Miller et al., 2014). Data repositories [e.g., Allen Brain Atlas (ABA), Stemformatics, Zenbu, CbGRITS, CDT-DB], and analysis tools offer means to inform unbiased classification strategies (Sato et al., 2008; Chen et al., 2013; Sunkin et al., 2013; Wells et al., 2013; Severin et al., 2014; Stein et al., 2014; van de Leemput et al., 2014; Ha et al., 2015). For example, statistical comparisons to ABA datasets from discrete brain regions have provided an important framework to evaluate the temporal and spatial identity of differentiated PSCs (Brennand et al., 2015; Pasca et al., 2015; Nayler et al., 2017). These tools and resources will be instrumental in elucidating factors that could be targeted to promote the maturation of cerebellar neurons beyond an embryonic stage.

While many of these datasets utilize whole population-based sampling, recent advances in microfluidic technologies and sequencing chemistry have led to robust methodologies for the sampling of single cells, which might reconcile a number of the current limitations (Jaitin et al., 2014; Habib et al., 2016). Fine-scale sampling of individual cells will allow population profiling and delineation of cell-type-specific markers and thus the identification and isolation of cells characteristic of their *in vivo* counterparts at specific developmental windows.

## Conclusion

In this article, we describe the major methods related to PSC-derived models of the cerebellum, including their provenance from early developmental biology studies. While the majority of differentiation protocols share some methodological overlap (primary neurulation, MHB specification, cerebellar patterning, and terminal differentiation/maturation), it appears that there are a number of distinct ways to achieve these goals. Waddington’s ‘Epigenetic Landscape’ theory (Waddington, 1939) has often been used to illustratively map the concept of lineage specification. This remains highly relevant in the context of cerebellar differentiation, given the varying protocols used to derive cerebellar neurons with seemingly different input requirements. Further understanding of the pathways and processes that govern cerebellar self-organization will be critical to producing the next generation of PSC-derived models of the developing cerebellum. Challenges will include the recapitulation of the laminar structure of the cerebellar cortex, with its distinct configuration of neuronal and non-neuronal cells, and highly stereotyped circuits. Moreover it will be interesting to demonstrate whether formation of cerebellar organoids into lobules, including the organization of cerebellar circuits in parasagittal compartments is possible. Another highly desirable goal, particularly in light of eventual potential therapeutic use of PSC-derived neuronal cells, is the elimination of xenogeneic culture methodologies. In the absence of current protocols this advanced for cerebellar differentiation, we have discussed several methodologies that are poised to advance the field, including the next generation of classification strategies.

One of the major motivations driving cerebellar PSC research forward is the potential for creating patient-specific models and better understanding cerebellar disorders (Watson et al., 2015). Recent examples highlight the exciting future direction of the field, with advances in creating iPSC models for spinocerebellar ataxia type 6 (SCA6) and Tuberous sclerosis complex (TSC), an autism spectrum disorder (Ishida et al., 2016; Sundberg et al., 2018). SCA6 patient-derived PCs showed vulnerability to triiodothyronine depletion, which could be suppressed with thyrotropin-releasing hormone and riluzole (Ishida et al., 2016), underscoring that patient-derived cells can be used to identify unknown early disease phenotypes, and as potential drug screening tools. Similarly, the disease phenotypes in TSC patient-derived PCs including abnormal differentiation, synaptic dysfunction and hypoexcitability, could be rescued by treatment with the mTOR inhibitor rapamycin (Sundberg et al., 2018).

Together, despite the remaining challenges in the field, and given the recent history of success in the field, we are extremely optimistic about the future of PSC-derived models in advancing our knowledge about cerebellar development and providing invaluable model systems to better understand and treat cerebellar disorders.

## Author Contributions

SN and EB wrote the manuscript. SN made and adapted figures with guidance from EB.

## Conflict of Interest Statement

The authors declare that the research was conducted in the absence of any commercial or financial relationships that could be construed as a potential conflict of interest.
