# Supplementary material for: The Use of Stem Cell-Derived Neurons for Understanding Development and Disease of the Cerebellum
Source: Front Neurosci. 2018 Sep 26;12:646. doi: 10.3389/fnins.2018.00646 (PMC6168705; doi:10.3389/fnins.2018.00646)
Supplement: Supplementary file 1 [file Data_Sheet_1.pdf]

Supplementary Material

The Use Of Stem Cell-derived Neurons For Understanding Development And Disease Of The Cerebellum

Samuel P. Nayler[1], Esther B. E. Becker[1*]

[1]University of Oxford, Department of Physiology, Anatomy and Genetics, Oxford, United Kingdom

*** Correspondence:** Esther B. E. Becker: esther.becker@dpag.ox.ac.uk

Reference	(Su et al., 2006)	(Salero & Hatten, 2007)	(Muguruma et al., 2010)	(Erceg et al., 2010)	(Srivastava et al., 2013)	(Wang et al., 2015)	(Higuera et al., 2017)
**Cells transplanted**	D9 Math1-GFP+ (mESC)	D24 *Pde1c-Egfp*-BAC (mESC)	D13+2 GAD-GFP Neph3+ (mESC)	D34+ MATH1-GFP+ (hESC)	D18+ DsRed (Math1-induced) (mESC)	D20 Neph3-GFP+ (hESC)	D20+(p2-6) Actin-GFP+ (mESC)
**Age mouse**	P2	P4-P6	E15.5	P7	P60	P0	P1 & Adult
**Injection site**	Cerebellum	EGL	Subventricular space of CP	EGL	Cerebellum	Cerebellum	Cerebellum (vermis & hemisphere)
**Cell number injected**	5,000-10,000	1,000	10,000	100,000	400,000	20,000	50,000 (P1) 75,000 (Ad)
**Volume injected**	2-4μl	1μl	2μl	100-200nl	2μl	1μl	1μl (P1) 1.5μl (Ad)
**Analysis**	5-14 days	1-15 days	1 & 4 wks	4 wks	1 wk	4 wks	25-30 days
**Survival rate post-injection**	0.1-0.3%		3%	14-18%			0.17% (P1) 2.8% (Ad)
**Result**	Migratory Math1+ cells, exhibiting leading and trailing processes associating with PF-like bundles. Expression of DCX, PAX6, GIRK2 and later GABAα6R.	*Pde1c-Egfp*-BAC cells migrated across ML into IGL. Interaction with MFs. Expression of ZIC2 and GABAα6R.	Grafted cells integrated into PCL and expressed L7 and Calbindin. Characteristic polarity with dendrites growing towards ML. VGluT2 expression along dendritic shafts.	Cells migrated across ML, past PCL and settled in IGL.	DsRed+ cells were detected in cerebellar lobules (mainly in ML) close to injection site. Cells displayed neuronal shape and expressed TUJ1. Started to colonize GL. Some neurons expressed GABAα6R.	2/12 mice showed GFP+/L7+ cells. Cells were detected in PCL with complex dendrite formation into ML, in ML and extra-cerebellar regions.	P1: 6/8 mice showed GFP labelling in cerebellar cortex. 9% of cells showed morphology of mature PCs and expressed Calbindin and Parvalbumin and received VGluT2-positive inputs.. Adult mice: 11/16 mice showed GFP labelling in cerebellar cortex (most in ML, some in PCL and GL or WM). 3.66% of GFP+ cells were Calbindin+ and showed mature PC morphology.

Supplementary Table 1. Summary of published experiments utilizing transplanted stem cell-derived cerebellar neurons to test integration and cell-fate commitment. Ad (Adult), CP (cerebellar plate), D (day of differentiation) ESC (embryonic stem cells), EGL (external granule layer), h (human), IGL (internal granule layer), GL (granule layer), m (mouse), ML (Molecular layer), PCL (Purkinje cell layer), P (postnatal day), p (passage number), PF (Parallel fiber), MF (Mossy fiber), wk (week), WM (white matter).

References

Erceg, S., Ronaghi, M., Zipancic, I., Lainez, S., Rosello, M.G., Xiong, C., et al. (2010). Efficient differentiation of human embryonic stem cells into functional cerebellar-like cells. *Stem Cells Dev* 19(11), 1745-1756. doi: 10.1089/scd.2009.0498.

Higuera, G.A., Iaffaldano, G., Bedar, M., Shpak, G., Broersen, R., Munshi, S.T., et al. (2017). An expandable embryonic stem cell-derived Purkinje neuron progenitor population that exhibits in vivo maturation in the adult mouse cerebellum. *Sci Rep* 7(1), 8863. doi: 10.1038/s41598-017-09348-1.

Muguruma, K., Nishiyama, A., Ono, Y., Miyawaki, H., Mizuhara, E., Hori, S., et al. (2010). Ontogeny-recapitulating generation and tissue integration of ES cell-derived Purkinje cells. *Nat Neurosci* 13(10), 1171-1180. doi: 10.1038/nn.2638.

Salero, E., and Hatten, M.E. (2007). Differentiation of ES cells into cerebellar neurons. *Proc Natl Acad Sci U S A* 104(8), 2997-3002. doi: 10.1073/pnas.0610879104.

Srivastava, R., Kumar, M., Peineau, S., Csaba, Z., Mani, S., Gressens, P., et al. (2013). Conditional induction of Math1 specifies embryonic stem cells to cerebellar granule neuron lineage and promotes differentiation into mature granule neurons. *Stem Cells* 31(4), 652-665. doi: 10.1002/stem.1295.

Su, H.L., Muguruma, K., Matsuo-Takasaki, M., Kengaku, M., Watanabe, K., and Sasai, Y. (2006). Generation of cerebellar neuron precursors from embryonic stem cells. *Dev Biol* 290(2), 287-296. doi: 10.1016/j.ydbio.2005.11.010.

Wang, S., Wang, B., Pan, N., Fu, L., Wang, C., Song, G., et al. (2015). Differentiation of human induced pluripotent stem cells to mature functional Purkinje neurons. *Sci Rep* 5, 9232. doi: 10.1038/srep09232.
